# The potential of PARP inhibitors in targeted cancer therapy and immunotherapy

**DOI:** 10.3389/fmolb.2022.1073797

**Published:** 2022-12-01

**Authors:** Jaromir Hunia, Karol Gawalski, Aleksandra Szredzka, Marcin J. Suskiewicz, Dominika Nowis

**Affiliations:** ^1^ Department of Immunology, Medical University of Warsaw, Warsaw, Poland; ^2^ Doctoral School, Medical University of Warsaw, Warsaw, Poland; ^3^ Laboratory of Experimental Medicine, Medical University of Warsaw, Warsaw, Poland; ^4^ Centre de Biophysique Moléculaire, Orléans, France

**Keywords:** PARP inhibitors, immune checkpoint inhibitors, immunotherapy, synthetic lethality, DNA repair, BRCA, cancer

## Abstract

DNA damage response (DDR) deficiencies result in genome instability, which is one of the hallmarks of cancer. Poly (ADP-ribose) polymerase (PARP) enzymes take part in various DDR pathways, determining cell fate in the wake of DNA damage. PARPs are readily druggable and PARP inhibitors (PARPi) against the main DDR-associated PARPs, PARP1 and PARP2, are currently approved for the treatment of a range of tumor types. Inhibition of efficient PARP1/2-dependent DDR is fatal for tumor cells with homologous recombination deficiencies (HRD), especially defects in breast cancer type 1 susceptibility protein 1 or 2 (BRCA1/2)-dependent pathway, while allowing healthy cells to survive. Moreover, PARPi indirectly influence the tumor microenvironment by increasing genomic instability, immune pathway activation and PD-L1 expression on cancer cells. For this reason, PARPi might enhance sensitivity to immune checkpoint inhibitors (ICIs), such as anti-PD-(L)1 or anti-CTLA4, providing a rationale for PARPi-ICI combination therapies. In this review, we discuss the complex background of the different roles of PARP1/2 in the cell and summarize the basics of how PARPi work from bench to bedside. Furthermore, we detail the early data of ongoing clinical trials indicating the synergistic effect of PARPi and ICIs. We also introduce the diagnostic tools for therapy development and discuss the future perspectives and limitations of this approach.

## Introduction

Cancer is a large group of diseases characterized by the uncontrollable growth of abnormal cells. There are 19.3 milion new cancer diagnoses each year worldwide, with an estimated 10 milion cancer-related deaths occurring in 2020, placing an enormous burden on healthcare systems (Sung et al., 2021).

Although the targeted cancer therapies have advanced noticeably in recent years, chemotherapy remains the most commonly used treatment in many kinds of cancer. Unfortunately, the side effects are inevitable, as chemotherapy is unable to differentiate malignant and non-malignant cells. Severe side effects of cancer treatment like vomiting (>90% of patients require antiemetics during the treatment), fatigue, generalized pain or gastrointestinal disturbances are common in patients (Lorusso et al., 2017) (Pearce et al., 2017). For this reason, the development of new therapies focuses on acting directly on cancer-specific targets, which, in theory, allows increased efficacy against cancer cells while minimizing side effects. Currently available treatments that meet the above requirements include small-molecule drugs (used for the targets inside the cells, such as proteasome inhibitors and signal transduction inhibitors) and monoclonal antibodies (designed to attach to specific targets on cancer cells). Generally, targeted therapies counter cancer through different mechanisms, such as inhibition of angiogenesis, blocking of the cell cycle, delivering cytotoxic substances directly into cancer cells etc.

One of the most dynamically developing targeted therapies are poly (ADP-ribose) polymerase (PARP) inhibitors (PARPi). Although this term in principle refers to inhibitors of any member of the PARP family of enzymes, most relevant for cancer therapy are those targeting PARP1 and PARP2, which are primarily involved in DNA repair (Lüscher et al., 2021). Therefore, inhibition of PARP1/2 results in genome instability that destroys cancer cells while allowing non-malignant cell survival. Since 2014, there are four PARPi approved for clinical use (olaparib, rucaparib, niraparib, talazoparib), indicated for the treatment of ovarian, fallopian tube and primary peritoneal carcinoma, HER2-negative breast cancer, metastatic pancreatic cancer and prostate cancer–especially (but not exclusively) when these cancers harbor breast cancer type 1 susceptibility protein 1 or 2 (BRCA1/2) mutations. In addition, there are many promising clinical trials at various stages that investigate other PARPi and their diverse indications. Moreover, PARPi are showing encouraging results in combined therapies, especially with immune checkpoint inhibitors (ICIs), holding great promise for many cancer patients. Although PARPi are thought to be relatively specific blocking agents and do not show frequent and severe side effects, PARP1/2 enzymes play many roles at different cell cycle stages. Therefore, drawing the interaction network of PARP1/2 in the cell is crucial for navigating the new possible therapies and determining possible side effects that may appear on the way. In this review, we try to place PARPi in that network, discuss recent combined therapies of PARPi and ICIs, and point out the possible future perspectives that come into view *en route*.

## PARP enzymes—Structure and mechanism of action

PARP proteins were discovered in the early 1960s by Chambon et al. (1963). Subsequent research showed that PARP family takes part in several cellular processes (Krishnakumar and Kraus, 2010), the most intriguing being the interaction with DNA (Nobori et al., 1989), which contributes to cellular recovery from DNA damage (Durkacz et al., 1980).

**FIGURE 1:**
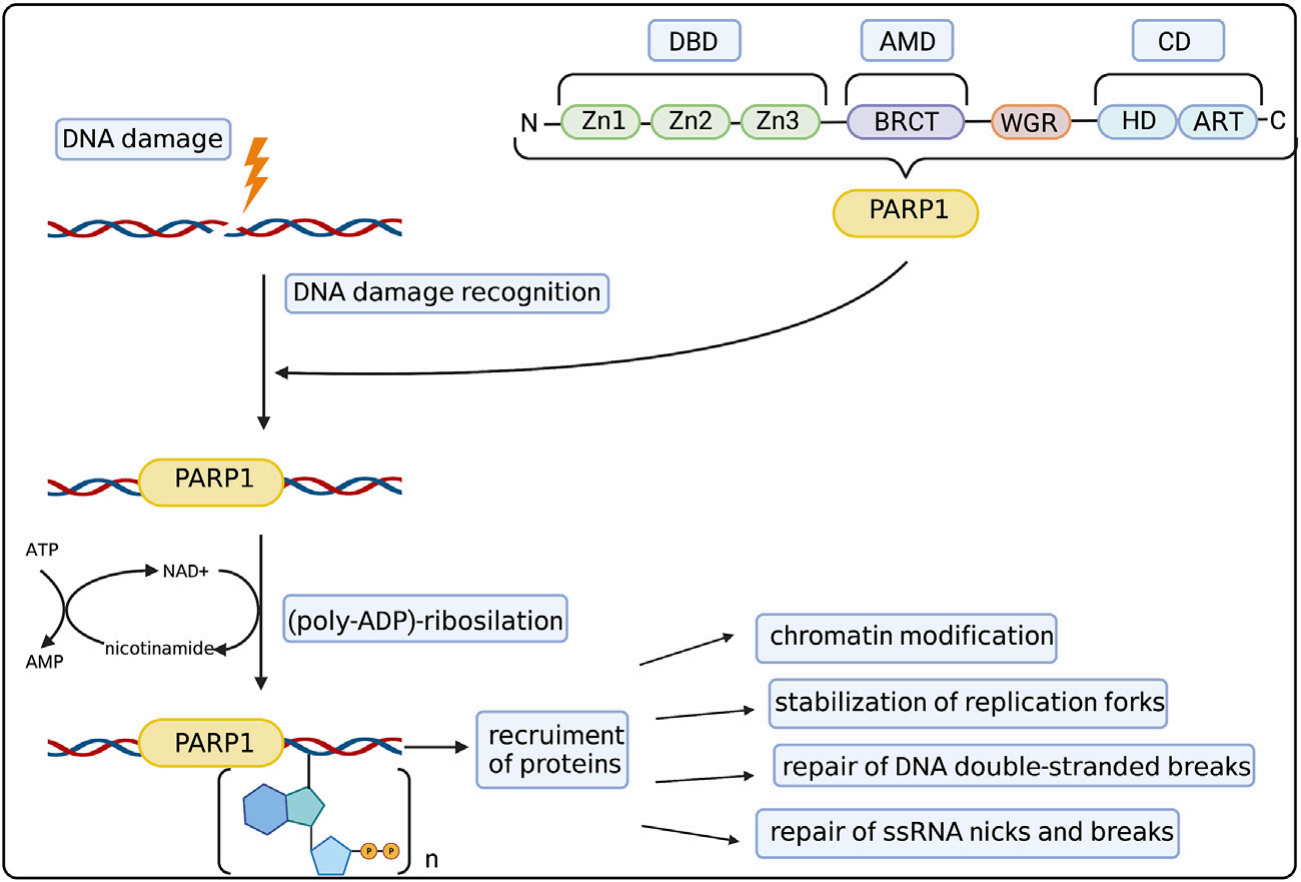
PARP1 structure and mechanism of action. PARP1 molecule consists of 3 main domains: DNA binding domain (DBD) with zinc fingers (Zn1-Zn3), Automodification Domain (AMD) with BRCA1 C-terminus (BRCT) subdomain and catalytic domain (CD) with helical domain (HD) and C-terminal ADP-ribosyl transferase (ART) subdomain. The WGR domain consists of the Trp-Gly-Arg motif. Such structure allows PARP1 to recognize and bind to the DNA damage site. Then it catalyzes the (poly-ADP)-ribosylation process, allowing the recruitment of auxiliary proteins responsible for chromatin modification, stabilization of replication forks, repair of DSBs and repair of ssDNA nicks and breaks. Created with BioRender.com.

First discoveries of possible functions of PARPs in the cell on the molecular level went hand in hand with dissecting its structure. To date the family of PARP proteins has 17 members that share structural and functional similarities while constituting a diverse and remarkable group of proteins. PARP family members are defined by their shared ADP-ribosyl transferase (ART) domain but otherwise differ in length and domain composition. PARPs generally function by modifying other proteins with single ADP-ribose units or poly (ADP-ribose) (PAR) chains (derived from nicotamide adenine dinucleotide–NAD^+^), which affect protein activity, interactions, localization and half-life. Moreover, PARP family members can also catalyse the reversible ADP-ribosylation of phosphorylated DNA and RNA ends, which was discussed by Groslambert et al. (2021). The 90% of total cellular PARylation is produced by PARP1—the most abundant and best studied member of the PARP family (Kameshita et al., 1984). The basic structure of the PARP1 molecule and its role in DNA repair was summarized in Figure 1. PARP1 consists of three main domains: the DNA Binding Domain (DBD) at the N-terminus, the central Automodification Domain (AMD), and the Catalytic Domain (CD) at the C-terminus (Hakmé et al., 2008). The most remarkable features of the DBD are the nuclear localization sequence (NLS) and three zinc fingers (Zn1/2/3). The latter are–together with the WGR motif in the CD–the primary sensors of DNA breaks, both Double Strand Breaks (DSBs) and Single Strand Breaks (SSBs) (Langelier et al., 2011), driving the collective assembly of PARP1 domains around the break in a way that stimulates the catalytic activity of this enzyme (Langelier et al., 2012) (Eustermann et al., 2015).

AMD is composed of a globular BRCA1 C-terminus (BRCT) motif and a linker region that comprises the main automodification (ADP-ribosylation) sites (Prokhorova et al., 2021b). The BRCT motif has been implicated in interactions with proteins and DNA alike (Yelamos et al., 2011) (Hottiger et al., 2010) (Rudolph et al., 2021a).

The main motif of the CD domain is called the signature motif of the ART subdomain. It is composed of six beta-sheets and one alpha-helix (Otto et al., 2005) and includes both an NAD^+^ binding pocket and the key catalytic residue, Glu988 (Schreiber et al., 2006). The regulatory helical domain (HD) consists of helices in the absence of DNA and becomes unfolded when PARP1 binds to damaged DNA (Dawicki-McKenna et al., 2015). The HD domain is thought to inhibit the access of NAD^+^ to the active site in the absence of DNA but allow NAD^+^ access upon DNA break-induced unfolding.

Upstream of the HD domain, the WGR (Trp-Gly-Arg) motif is the key regulator of catalytic activity in response to DNA damage by interacting with DNA (Langelier et al., 2012).

In addition to PARP1, two other PARPs have been implicated in DNA damage response (DDR): PARP2 and PARP3. These paralogues are shorter than PARP1, lacking the N-terminal portion comprising zinc finger and BRCT motifs. The WGR motif of PARP2 and PARP3 is responsible for DNA binding as well as sensing the nature of DNA breaks, especially phosphorylation (Langelier et al., 2014). In contrast, PARP1 is relatively insensitive to the phosphorylation state of DNA breaks that it binds to and is activated by (Obaji et al., 2018) (Bilokapic et al., 2020). Specifically in the case of PARP2, the WGR domain is apparently capable of simultaneously binding to two blunt DNA ends, possibly in order to bridge them for subsequent ligation (Gaullier et al., 2020)

The product of PARP activity, the PAR chain, is a large, negatively charged polymer composed of ADP-ribose monomers connected through glycosidic ribose-ribose bonds. The chains can be linear or branched and are typically attached to protein substrates. PARPs can also modify proteins with only a single ADP-ribose unit (mono (ADP-ribose) or MAR. PAR and MAR modification occurs mostly on Ser, Glu, and Asp residues in proteins, and can be enzymatically removed by specific ADP-ribosyl hydrolases (Palazzo et al., 2018) (Larsen et al., 2018) (Hendriks et al., 2019).

## PARP1 in DNA repair

Enzymatic DDR mechanisms correct the damage that occurs spontaneously in the cell during metabolic changes under the influence of both exogenous and endogenous (physical and chemical) factors. These mechanisms act during different phases of the cell cycle, both during replication and during the intervals between divisions. If the defects are not recognized and removed, they may lead to permanent changes in DNA and mutations in subsequent cycles. Due to the variety of factors influencing DNA integrity, multiple repair mechanisms have developed in cells. The most significant DDR pathways include:

1) Nucleotide excision repair (NER), which is a highly conserved DDR pathway that corrects a wide range of genomic lesions, especially double helix-distorting bulky lesions (Spivak, 2015),
2) Base excision repair (BER), occurring mainly during the G1 phase of the cell cycle, repairing forms of damage that do not significantly distort the DNA helix (Dianov and Hübscher, 2013). To note, the BER pathway generates SSBs, which are the most common lesions occurring in the cell (Caldecott, 2008). They are also generated by various endogenous or exogenous DNA-damaging agents, such as ionizing radiation, free radicals, topoisomerase I (TOP1) (Caldecott, 2008). SSBs may cause the blockade or collapse of DNA replication forks during the S phase, leading to the formation of DSBs (Kouzminova and Kuzminov, 2006) (Kuzminov, 2001). SSBs in non-proliferating cells may stall RNA polymerase progression during transcription, leading to cell death (Bendixen et al., 1990). SSBs are repaired in the SSB Repair (SSBR) process (Dianov and Hübscher, 2013), which is sometimes considered as a subpathway of BER (Abbotts and Wilson, 2017). SSBR is orchestrated by X-ray repair cross complementing protein 1 (XRCC1) and PARP1 as the key proteins and generally consists of SSB detection (by PARP1), DNA end processing, DNA gap-filling and DNA ligation (Abbotts and Wilson, 2017).
3) Mismatch repair (MMR) removes errors that were not corrected by DNA polymerases during replication (Kunkel and Erie, 2015),
4) Homologous recombination (HR) dominates the late S and G2 phases (Branzei and Foiani, 2008). Upon damage detection, BRCA1, BRCA2 and a partner and localizer of BRCA2, PALB2, recruit the RAD51 recombinase, which is the end effector in HR that performs DSB repair using a homologous chromatid as a template. DSBs are especially dangerous for the cell and can easily lead to apoptosis. In addition to HR, they can also be repaired *via* different pathways depending on cell cycle phase in which they occur (Chapman et al., 2012).
5) Non-homologous end joining recombination (NHEJ) is an alternative pathway for repairing DSBs, preferred during the G1 phase of the cell cycle. (Branzei and Foiani, 2008) (Lee and Dutta, 2021). DSBs are recognized by the Ku70-Ku80 heterodimer, which allows recruitment of DNA-dependent protein kinase (DNA-PK), DNA ligase 4 (LIG4) and the X-ray repair cross-complementing protein 4 (XRCC4) factor. These proteins activate the process, stabilize DNA and orientate it during LIG4-mediated ligation. If the ends of the DNA are incompatible, they can be adjusted for ligation by the nuclease Artemis or by DNA polymerase mu (Polμ), DNA polymerase lambda (Polλ), and terminal deoxynucleotidyl transferase (TdT) polymerases. NHEJ can also be divided into two main pathways of common characteristics: classical (cNHEJ) and alternative (aNHEJ). While cNHEJ comprises most of the features described above and attributed to NHEJ in general, aNHEJ is a more recently discovered mechanism of DSB repair, serving as a less efficient backup reaction. Its activity has been noticed during cNHEJ deficiency or impairment (Zhao et al., 2020).

**FIGURE 2:**
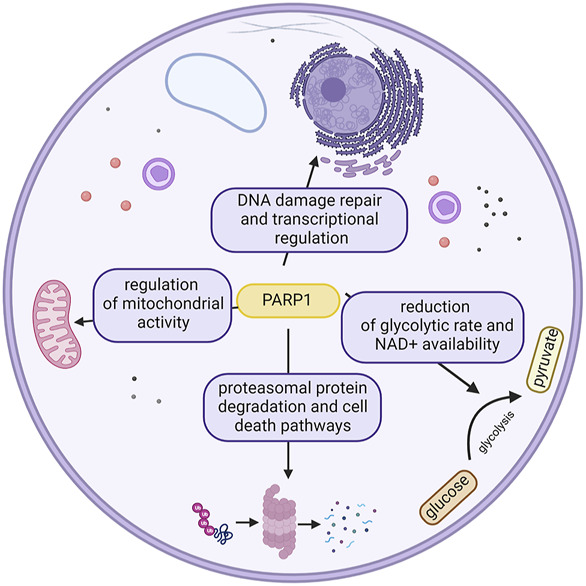
Different roles of PARP1 in the cell. This ubiquitousness defines the potential effects of its inhibition. Created with BioRender.com.

In this complex scenery of proteins engaged in the diverse repertoire of DNA repair pathways, one of the most intriguing characters belong to the PARP family, which is known to act in various pathways of the cell (Figure 2); however, their most remarkable feature is undoubtedly initiation of the DDR pathway (Ray Chaudhuri and Nussenzweig, 2017) (Langelier et al., 2014).

In the DDR process, PARP1 is involved in three general steps: 1) detection of DNA damage, 2) recruitment of co-factors and 3) regulation of biochemical activities. Initially, PARP was mostly known to be involved in the BER and SSBR pathways, involving such factors as XRCC1 and DNA ligase 3 (LIG3) (Fisher et al., 2007) (El-Khamisy, 2003) (Dantzer et al., 2000). Later studies showed the role of PARP1 in NER (Pines et al., 2012) (Robu et al., 2013), cNHEJ (Luijsterburg et al., 2016) and aNHEJ (Mansour et al., 2010), microhomology-mediated end joining (MMEJ) (Dutta et al., 2017), HR (Hochegger et al., 2006) (Hu et al., 2014), MMR (Liu et al., 2011), and maintenance of replication fork stability (Ronson et al., 2018) (Yang et al., 2004). Notably, recent publications point out the connection between PARP1 and Okazaki fragments (Hanzlikova et al., 2018) (Vaitsiankova et al., 2022).

Regarding the function in replication fork stability, PARP1 and PARP2 act in concert to detect disrupted replication forks, recruit the repair protein Mre11, stimulate recombination repair and restart replication (Bryant et al., 2009). Mre11 is activated by PARP at the stalled fork and restarts the Mre11-dependent replication and recombination (Kim et al., 2005).

The recruitment of different binding proteins to PARP1 is possible because of the zinc fingers 1–3. However the presence of zinc finger domains seems not to be necessary, as PARP2 and PARP3 are also able to recognize specific DNA breaks featuring 5’ phosphate groups, despite lacking the zinc finger domains. This process also leads to DDR initiation (Langelier et al., 2014). In this case the WGR domain, present also in PARP1, allows PARP2 and PARP3 to interact with DNA. Also the N-terminus of PARP2 manifests DNA-binding activity. It plays a role in the activation of PARP2 on SSBs (Riccio et al., 2016). PARP1 and PARP2 molecules remain mostly monomeric while in free form, but they form temporary dimer formations while binding to nicked sites or gaps (Eustermann et al., 2011).

DNA damage sensing is a clocklike mechanism and requires precise mechanism of break site detection as well as fast transport to the damaged site on DNA and between such sites. One recent speculative model for PARP1’s movement along DNA is called the ‘monkey bar’ mechanism (inter-segment transfer) (Rudolph et al., 2018). In this mechanism, PARP1 moves from one DNA break to the next in such a way that it always holds on to at least one DNA break, like a monkey that is holding a branch with one hand until it grasps the next branch with the other hand. This is due to the fact that binding to a new DNA break is required in order to stimulate release from the previous break. This mechanism is thought to enhance the ability of PARP1 to effectively scan genome and detect the level of DNA damage (Rudolph et al., 2018). The conformational change of the WGR domain was shown to be crucial in this process (Rudolph et al., 2020).

As described above, engagement with a DNA break through zinc finger domains and WGR is also key to the allosteric activation, which is mediated by the destabilization of the HD subdomain (Dawicki-McKenna et al., 2015) (Langelier et al., 2008).

As the PAR balance is crucial for the cell, the processes of PARylation and dePARylation are tightly controlled to maintain the equilibrium (D’Amours et al., 1999) (Fouquerel et al., 2014). PAR removal is carried out by poly (ADP-ribose) glycohydrolase (PARG) and ADP-ribosyl-acceptor hydrolase 3 (ARH3) (Rack et al., 2020) (Fontana et al., 2017) PARG and ARH3 suppression were noted to be synthetically lethal due to the increase in PARylation. (Prokhorova et al., 2021a).

PARP enzymes are able to covalently modify various targets, like transcription factors (involving transcriptional repressor CTCF, activator protein 1 (AP1), yin yang 1 (YY1), nuclear factor kappa-light-chain-enhancer of activated B cells (NF-kB), nuclear enzymes (aurora B kinase) (Kraus, 2008) (Monaco et al., 2005), and histone H1, H2A and H2B, regulating the chromatin structure (Kim et al., 2005). PARPs also tend to modify themselves (automodification), which is particularly well established for PARP1 that ADP-ribosylates a set of conserved residues in the AD. PARylation is known to play an important role in the epigenetic regulation of chromatin structure, as well as gene expression, under physiological conditions, with the maintenance of DNA integrity (Caiafa et al., 2009). On the epigenetic level, histone PARylation factor 1 (HPF1) is one of the most essential proteins interacting with PARP1 and PARP2 enzymes (Gibbs-Seymour et al., 2016). Recent findings of Suskiewicz et al. describe the role of HPF1 as a molecule complementing the PARP1/2 active site essential for the addition of ADP-ribose moieties in the DDR process (Suskiewicz et al., 2020). It turns out that the most common cellular acceptor of ADP-ribosylation–protein serine residues–cannot be efficiently targeted by PARP1 or PARP2 alone, and HPF1 must be present.

Besides ribosomal and nuclear DNA, PARP1 was also reported to be involved in the epigenetic regulation of mtDNA repair and transcription (Lapucci et al., 2011).

## PARP1 in metabolic regulation

PARP enzymes are NAD + consumers with the capacity to globally affect the cellular energy pool and metabolic state, potentially having dramatic cellular consequences. Thus, their enzymatic activity is tightly controlled (Andrabi et al., 2014). PARP1 is also known to influence mitochondrial function and oxidative metabolism. Studies on a 129/SvImJ mouse knockout showed that PARP1 deletion led to increased food intake (Devalaraja-Narashimha and Padanilam, 2010). Further investigation showed the reduction in the glycolytic rate, which has been linked to a reduction in NAD+ availability over the years (Bai et al., 2011). PARP1 over-activation was shown to be able to reduce hexokinase activity, thus resulting in global carbohydrate metabolism perturbations (Houtkooper et al., 2010) (Andrabi et al., 2014)

## PARP1 in cell death

PARPs have been reported to take part in several kinds of cell death pathways. At moderate levels of DNA damage apoptosis is the key feature, with Caspase3 and Caspase7 cutting PARP1 molecule into 2 fragments after D214, between first two zinc finger domains and Zn3 (Soldani and Scovassi, 2002) (Germain et al., 1999) (Kaufmann et al., 1993) (Tewari et al., 1995). At highly elevated levels of DNA damage, PARP1 overactivation is observed, resulting in necrotic cell death (caused by depletion of NAD^+^ and ATP) (Sosna et al., 2014). Meanwhile PARP1 detecting low levels of DNA damage steers the fate of cells into survival and DNA repair (Bouchard et al., 2003).

PARP1, along with apoptosis inducing factor (AIF), has been suggested to play a crucial role in another kind of caspase-independent cell death, parthanatos (Yu et al., 2006). Parthanatos was thought to be the ultimate effect of PARP overactivation leading to increased NAD^+^ consumption and subsequent loss of ATP (Ha and Snyder, 1999), until the decrease of NAD^+^ and ATP was associated with glycolysis inhibition through PAR chain binding on the PAR-binding motif (PBM) of hexokinase (Andrabi et al., 2014) (Fouquerel et al., 2014). It can be specifically observed in stroke, diabetes, and Parkinson disease (Andrabi et al., 2008).

Besides this, PARP1 is engaged in DNA damage-dependent autophagy (Muñoz-Gámez et al., 2009) and shows a cytoprotective role in oxidative stress-induced necrotic cell death. PARP1 also stimulates the unfolded protein response (Shoab Mansuri and Singh, 2014).

## PARP1 in cancer

PARP1 may facilitate HR by recruitment of such proteins as ataxia telangiectasia mutated (ATM), nibrin (NBS1), and Mre11 to sites of DSB of DNA (Haince et al., 2008), however its major role in the HR repair involves localization of BRCA1/2. BRCA1 is involved in the surveillance of DNA damage and transduction of DNA repair responses, while BRCA2 is directly engaged in DNA DSBR *via* modulation of Rad51 by HR (Tutt and Ashworth, 2002). Their mutations are able to prevent DNA repair mechanisms and increase the risk of malignances (Hennessy et al., 2010).

PARP1 overexpression has been demonstrated in many cancers, which indicates its importance in cancerogenesis and may be an independent prognostic factor. In colorectal cancer level of PARP1 expression was positively correlated with tumor size and histopathological features according to TNM classification system (Nosho et al., 2006). Increased levels of PARP1 have also been observed in breast cancer (Siraj et al., 2018), gastric cancer (Afzal et al., 2019), ovarian cancer (Kummar et al., 2012), pancreatic cancer (Xu et al., 2019), and liver cancer (Li et al., 2017) The dependence of prostate cancer on PARP1 activity was shown in the reduction of AR to PARP1 inhibition (Espinoza, 2013).

PARP1 is also relevant for diseases other than cancer. There is evidence that it is associated with the pathogenesis of diseases such as rheumatoid arthritis (García et al., 2006) (Li et al., 2016), chronic gastritis (Lee et al., 2016), acute and chronic inflammatory bowel disease (Sánchez-Fidalgo et al., 2007), allergic responses and asthma (Ghonim et al., 2015) (Oumouna et al., 2006), oxidative/nitrosative stress following infarction-reperfusion and septic shock (Soriano et al., 2006) (Kang et al., 2010) (Pazzaglia and Pioli, 2019).

## PARP inhibitors - Rationale

Considering PARP1’s role in the DDR and having in mind that genome instability is one of the hallmarks of cancer, the idea of developing PARPi arose in oncology studies. Almost 3 decades ago, Satoh et al. (1994) showed that as much as 90% of PARP activity must be stopped to impair the DNA repair process. Hence, potential PARPi have to manifest both high specificity and effectiveness, to bear a potential for clinical application (Bryant et al., 2005) (Farmer et al., 2005) (Calvert and Azzariti, 2011) (Plummer et al., 2008) (Calabrese et al., 2003). The PARPi currently developed and used in clinics are mainly active against PARP1 and its closest homologue, PARP2, but only weakly against other PARPs (Rudolph et al., 2022). The attempts to develop PARPi led to two different approaches: 1) targeting cells which are predisposed to cell death when PARP activity is lost; 2) searching for a combination therapy with a different type of DNA-damaging agents (Papeo et al., 2013). First of these ideas is presented by PARPi’s role in the potential therapy of cells with deficiency in the *BRCA1/2*-dependent HR pathway–a process known as a synthetic lethality (Lord and Ashworth, 2017). This approach has medical implications, establishing PARPi as potential drugs in *BRCA1/2*-mutated (*BRCA1/2*m) cancers, as has been validated both *in vitro* and *in vivo* (Bryant et al., 2005) (Farmer et al., 2005). Later on, PARPi were successfully translated into clinical treatment of *BRCA1/2*m cancers such as breast (Robson et al., 2017) and ovarian cancer (Coleman et al., 2019). To date, a large number of research studies, trying to apply PARPi in other cancers, is in progress (Table 2).

The antitumor potential of PARPi—which has been widely observed and confirmed in multiple studies—is an intensively explored area today, so numerous hypotheses are emerging, but the exact mechanism of action of PARPi is still not fully understood.

The first described mechanism of PARPi action was associated with the well-known role of PARP1 in SSBR. It linked PARP1 inhibition with blocking this pathway and therefore triggering a process known as synthetic lethality (Bryant et al., 2005). It occurs when the combination of at least two genetic or molecular events results in a death of a cell or an organism (Nijman, 2011). PARP1 hyperactivation was noted in HR-defective cells, making them more sensitive to PARPi (Gottipati et al., 2010). However, later studies investigating the genetic knockout (Horton et al., 2014), or molecular silencing with siRNA (Patel et al., 2011) of a marker molecule of PARP1 activity—XRCC1, showed confounding results depending on whether it was genetic or molecular context of depletion. Disfunctional SSBR could lead to accumulation of DSBs, which, ultimately, can only be repaired by HR or NHEJ pathways, with NHEJ being much more error-prone, while HR needs *BRCA1/2* genes to proceed (Isono et al., 2017) (Holloman, 2011) (Lieber, 2008) (Ashworth, 2008). In *BRCA1/2*-deficient tumors, the only way to repair the DSB upon PARP1 inhibition is NHEJ, which, due to its potency to generate mutations, leads to a genomic catastrophe. In addition to the SSBR connection, the synthetic lethality between PARPi and HR deficiencies could also be explained by the roles of both PARP1 and BRCA1/2 in replication fork stability and restart (Bryant et al., 2009) (Koppensteiner et al., 2014) or DDR.

Besides the fact that PARP1 inhibition favours the NHEJ pathway by blocking the alternative HR, the interactions between PARP1 and NHEJ are much more complex. It was suggested that PARPi treatment increases the phosphorylation of DNA-dependent protein kinase (DNA-PK) substrates and therefore increases NHEJ activity (Patel et al., 2011). Indeed, inhibition of PARP1 ultimately blocks its interactions with Ku70 and Ku80, which are negative regulators of this pathway, therefore leading to increase in NHEJ activity (Wang et al., 2006) (Hochegger et al., 2006) (Paddock et al., 2011) (Yang et al., 2018).

Recent studies suggest that PARP inhibition-associated effects may be the result of DSBs occurring as a result of high-speed replication (Maya-Mendoza et al., 2018) (Quinet and Vindigni, 2018). This might in turn cause the accumulation of cytotoxic replication-associated single stranded DNA (ssDNA) gaps (Cong et al., 2021), subsequently leading to reversal of stalled replication forks. This theory is relatively unexplored and new; however, there is existing evidence supporting this mechanism of action of PARPi (Cong et al., 2021). If PARPi function solely by blocking the action of PARP1, then their effect should not be greater than that of the genetic deletion of the PARP1 gene. However, several authors have reported that inhibiting PARP1 is more cytotoxic than deleting it, even in chicken cells, which lack the PARP2 orthologue that PARPi inhibit in addition to PARP1 in human cells (Murai et al., 2012) (Patel et al., 2012) (Pettitt et al., 2013). This implies that PARP1 that is blocked by PARPi not only fails to perform its physiological role(s) but also acquires a new, toxic function. This line of reasoning—together with the observed accumulation of PARP1 on chromatin upon PARPi treatment—led to the hypothesis of PARP trapping proposed by Murai et al. (2012), whereby inhibited PARP1 persists on DNA damage and actively interferes with vital cellular processes. In general, PARP1 is thought to undergo a cycle whereby it associates with DNA breaks, becomes catalytically activated, and then modifies both other substrates and itself. Automodification of PARP1 prevents PARP1’s association with a DNA break, so PARP1 dissociates from DNA and eventually becomes dePARylated by PARG, terminating the cycle. This cycle was elaborated early on in publications by Ferro and Olivera (Ferro and Olivera, 1982), Zahradka and Ebisuzaki. (1982) and, especially, Satoh and Lindahl. (1992). Murai et al. proposed that interference with PARP1’s ability to automodify could explain the trapping effect of PARPi (Murai et al., 2012). Additionally, these authors hypothesized that some PARPi might have an allosteric effect, making PARP1 more tightly associated with DNA independent of automodification inhibition, but this has not been confirmed for the current clinical PARPi in recent publications (Zandarashvili et al., 2020) (Rudolph et al., 2022) (Hopkins et al., 2015) (Xue et al., 2022). Another revision to Murai et al.‘s model was recently offered by Shao et al. (Shao et al., 2020), who showed that PARP1, even in the “trapped state,” is not physically stalled on DNA but rather undergoes constant exchange. Nonetheless, PARP1 molecules that are exchanging on DNA might, considered as a population, could effectively outcompete other factors from binding to DNA, e.g., those required for an efficient replication fork restart or those engaged in DNA repair progression etc., explaining in part the particularly toxic effect of PARP inhibition.

Although in cancer therapies PARP trapping is a desired mechanism, it is an obstacle in conditions characterized by PARP hyperactivation, i.e. neurodegenerative diseases or ischemia-reperfusion injury. An interesting solution might be group of PROTAC PARP degraders, which are able to inhibit PARP1 without trapping, mimicking PARP1 depletion and protecting the cell from genotoxic stress-induced cell death (Wang et al., 2019a).

Finally, the role of PARP1 in histone modification and chromatin structure regulation is widely discussed and briefly reviewed here. Discovering these actions of PARP1 naturally leads to formation of a new hypothesis encompassing the epigenetic aspect of PARP1. Inhibition of PARP1 could also lead to inhibition of important oncogenes that are controlled by PARP1-dependent histone modification. This theory was already explored in Ewing sarcoma (Brenner et al., 2012) and *BRCA1/2*-deficient breast cancer patients (Kim et al., 2019).

## PARP inhibitors—In clinical use

Based on their presumed mechanism of action, PARP inhibitors are currently approved for the treatment of breast, ovarian, pancreatic, and prostate cancers carrying *BRCA1/2* mutations. However, their application is limited by the relatively low percentage of *BRCA1/2*m occurring in 10%–15% of breast and ovarian, 4%–7% of pancreatic and 1.5% of prostate tumors (Bryant et al., 2005) (Iqbal et al., 2012). On the other hand, recent studies showed PARPi might be effective in tumors which do not carry any *BRCA1/2*m, but have different alternative HR deficiencies or other DDR gene alterations (Bryant et al., 2005) (Turner et al., 2008) (Jonsson et al., 2019). Moreover, tumor cells face both oxidative and replicative stress, which, as studies suggest, makes them more sensitive to blocking DNA repair pathways i.e. using PARPi monotherapy (Majuelos-Melguizo et al., 2015) (Schoonen et al., 2017) (Michelena et al., 2018). These studies broaden the possible application of PARPi in tumor therapies.

All four PARPi approved for treatment (olaparib, talazoparib, niraparib, and rucaparib) share similarities on the molecular level. The common element of their chemical structure is an aromatic system that mimics nicotinamide (a part of NAD^+^), allowing PARPi to compete with NAD^+^ for PARP binding. The precise way in which a given PARPi engages with the NAD^+^-binding pocket determines how efficient it is at outcompeting NAD^+^ and thus blocking the catalytic activity (including automodification). Additionally, PARPi extend to a varying degree towards the HD domain and can affect the position and folding of HD helices. Since the HD is allosterically coupled—*via* the WGR—with DNA breaks, PARPi can in principle allosterically modulate DNA binding, in addition to affecting it indirectly *via* PARP automodification inhibition. In terms of the allosteric effects, PARPi can be divided into threegroups: 1) promoting allosteric retention on DNA (including several PARPi not yet used in the clinic), 2) allostery-neutral drugs (olaparib, talazoparib), and 3) drugs having an allosteric pro-release effect (rucaparib, niraparib, veliparib), as proposed (Zandarashvili et al., 2020). However, later studies of Lunger’s group (Rudolph et al., 2022) (Rudolph et al., 2021b) showed the affinity of PARPi depends rather on known trapping properties than the allosteric effect, which is additional and either neutral or negative in all existing clinical PARPi. By determining both the affinity for the NAD^+^ pocket and any potential allosteric effects, the molecular structure of PARPi is key to determining their molecular properties including trapping potential. Here, we briefly introduce the quartette of PARPi, more detailed description covering the FDA/EMA recommendations can be found in Table 1.

**TABLE 1:** History of PARPi in the clinic–European Medicines Agency (EMA) and Food and Drug Administration (FDA) approvals. Full access to the research studies description is available on ClinicalTrials.gov.

PARP inhibitor	Year of approval	Approving organization	Indication	Mutational requirement	Relevant studies
Olaparib	2014	FDA, EMA	Advanced ovarian carcinoma	Germline *BRCA1/2*m	NCT0107662 (Kaufman et al., 2015)
	2017	FDA, EMA	Recurring ovarian, fallopian tube and primary peritoneal carcinoma	independent of *BRCA1/2* mutational status	SOLO-2 and Study 19 (Pujade-Lauraine et al., 2017)
	2018	FDA	HER2 negative breast cancer	*BRCA1/*2m	OlympiAD (Robson et al., 2017)
	2019	EMA	HER2 negative breast cancer	*BRCA1/2*m	OlympiAD (Robson et al., 2017)
	2018	FDA	First-line treatment of advanced ovarian, fallopian and primary peritoneal carcinoma	Germline *BRCA1/2*m	SOLO-1 (Moore et al., 2018)
	2019	EMA	First-line treatment of advanced ovarian, fallopian and primary peritoneal carcinoma	Germline *BRCA1/2*m	SOLO-1 (Moore et al., 2018)
	2019	FDA	Metastatic pancreatic cancer	*BRCA1/2*m	POLO (Golan et al., 2019)
	2020	FDA	First-line treatment of advanced ovarian, fallopian and primary peritoneal carcinoma in combination with bevacizumab	HRD-positive, complete or partial chemotherapy response	PAOLA-1 (Ray-Coquard et al., 2019)
	2020	FDA	Metastatic castration-resistant prostate cancer	HRD-positive	PROfound (de Bono et al., 2020)
	2022	FDA	Deleterious or suspected deleterious high-risk early breast cancer that have been treated with adjuvant or neoadjuvant chemotherapy	Germline *BRCA1/2*m, HER2-negative	OlympiaA (Tutt et al., 2021)
Rucaparib	2016	FDA	Advanced ovarian carcinoma, following multiple chemotherapy treatments	*BRCA1/2*m	ARIEL2 and Study 10 (Oza et al., 2017)
	2018	EMA	Advanced ovarian carcinoma, following multiple chemotherapy treatments	*BRCA1/2*m	ARIEL2 and Study 10 (Oza et al., 2017)
	2018	FDA	Recurring ovarian, fallopian tube and primary peritoneal carcinoma	independent of *BRCA1/2* mutational status	ARIEL3 (Coleman et al., 2017)
	2019	EMA	Recurring ovarian, fallopian tube and primary peritoneal carcinoma	independent of *BRCA1/2* mutational status	ARIEL3 (Coleman et al., 2017)
	2020	FDA	Metastatic castration-resistant prostate cancer	*BRCA1/2*m	TRITON2 (Abida et al., 2019)
Niraparib	2017	FDA, EMA	Recurring ovarian, fallopian tube and primary peritoneal carcinoma	complete or partial chemotherapy response	ENGOT-OV16/NOVA (Mirza et al., 2016)
	2019	FDA	Recurring ovarian, fallopian tube and primary peritoneal carcinoma	HRD-positive, independent of chemotherapy response	QUADRA (Moore et al., 2019)
	2020	FDA, EMA	Advanced ovarian carcinoma and primary peritoneal carcinoma	independent of biomarker status, complete or partial chemotherapy response	PRIMA (González-Martín et al., 2019)
Talazoparib	2018	FDA, EMA	Advanced or metastatic HER2-negative breast cancer	Germline *BRCA1/2*m	EMBRACA (Ettl et al., 2018)

Olaparib (Lynparza) was the first drug to be approved in 2014 by EMA and FDA in clinical use as monotherapy for the treatment of advanced germline *BRCA1/2*m ovarian cancer (Wiggans et al., 2015) (Deeks, 2015).

Since the first approval of olaparib in clinic, several next-generation PARPi (i.e. talazoparib, niraparib, and rucaparib) have been tested in clinical trials (Murai et al., 2012). Talazoparib (Talzenna), a drug targeting both PARP1 and PARP2 (Shen et al., 2013), was approved in 2018 for the treatment of the germline *BRCA1/2*m-advanced or metastatic *HER2*-negative breast cance (Ettl et al., 2018) (Hoy, 2018). Talazoparib has an exceptionally high affinity for PARP1 (up to 100 fold higher trapping efficiency than olaparib) and therefore requires very low concentration to produce an effect that would require a considerably higher concentration of olaparib. Nevertheless, since 2018, talazoparib has not been approved for any further treatment. The most selective PARP1 and PARP2 inhibitor in clinical use is considered to be niraparib (Thorsell et al., 2017). Niraparib (Zejula) was approved in 2017 in the US and the EU for maintenance treatment of reoccurring ovarian, fallopian, and primary peritoneal carcinomas, regardless of their *BRCA1/2*m status, in patients that show complete or partial response to chemotherapy (Mirza et al., 2016) (Del Campo et al., 2019) (Scott, 2017).

Unlike olaparib and niraparib, rucaparib (Rubraca) inhibits PARP3 in addition to PARP1 and PARP2. As PARP3 has been suggested to activate the enzymatic activity of PARP1 in the absence of DNA, rucaparib’s ability to inhibit PARP3 may potentiate its effects compared to olaparib or niraparib (Loseva et al., 2010). It was first approved by FDA in 2016 for somatic and germline *BRCA1/2*m advanced ovarian carcinomas in patients following multiple chemotherapy trials (Oza et al., 2017) (Syed, 2017).

To date, all four clinically-approved PARPi have been tested in various clinical trials to broaden their application into different cancers (Table 2). The highly specific mechanism of action of PARPi does not exclude their toxicity, especially considering PARPs comprehensive role and its omnipresence in the cell. PARPi show side effects that are characteristic for the class and for each drug separately that should be considered while making clinical decisions (LaFargue et al., 2019). (Supplementary Table S1).

**TABLE 2:** List of selected PARP inhibitor monotherapy trials. Full access to the research studies description is available on ClinicalTrials.gov.

PARP inhibitor	Tumor	Phase	Status	Trial ID (NCT number)
Olaparib	Pancreatic acinar cell carcinoma	2	Recruiting	NCT05286827
	Castration-resistant prostate adenocarcinoma	2	Recruiting	NCT04951492
	Mesothelioma	2	Recruiting	NCT04515836
	BRCAwt platinum-sensitive recurrent ovarian cancer	2	Recruiting	NCT04091204
	Metastatic renal cell carcinoma	2	Recruiting	NCT03786796
	Oral squamous cell carcinoma	1, 2	Recruiting	NCT03085147
Rucaparib	Metastatic castration-resistant prostate cancer, epithelial ovarian cancer, fallopian tube cancer, peritoneal cancer	3	Enrolling by invitation	NCT04676334
	Metastatic endometrial cancer	2	Recruiting	NCT03617679
Niraparib	Recurrent gliomas	2	Recruiting	NCT05297864
	Endometrial cancer	Early 1	Not yet recruiting	NCT05289648
	Metastatic breast cancer in germline PALB2m carriers	2	Not yet recruiting	NCT05232006
	Leiomyosarcoma	2	Not yet recruiting	NCT05174455
	Advanced PALB2m tumors	2	Recruiting	NCT05169437
	Tumors metastatic to central nervous system	2	Recruiting	NCT04992013
	HPV-negative squamous cell carcinoma of head and neck	2	Recruiting	NCT04681469
	Ovarian cancer	2	Recruiting	NCT04507841
	Castration-resistant prostate adenocarcinoma	2	Recruiting	NCT04288687
	Metastatic melanoma with genetic HR mutation	2	Recruiting	NCT03925350
	Metastatic esophageal cancer, gastric cancer	2	Recruiting	NCT03840967
	Pancreatic cancer	2	Recruiting	NCT03601923
Talazoparib	Acute myeloid leukemia, myelodysplastic syndrome	1	Recruiting	NCT03974217
	Malignant solid neoplasms (breast carcinoma, gastric carcinoma, ovarian carcinoma etc.)	2	Recruiting	NCT04550494
	Ovarian cancer, fallopian tube cancer	1	Recruiting	NCT04598321

## PARP inhibitors—Resisitance

One of the biggest obstacles, which must be faced in the successful translation of PARPi into the clinic as an anti-cancer therapy, is frequently observed tumor resistance. The most well-known mechanism of DNA repair developed by tumor cells is the restoration of HR activity (Noordermeer and van Attikum, 2019). The mechanisms responsible for this are: recreation of BRCA1/2 activity, observed in clinical trials in patients with *BRCA1/2*m cancers (Kondrashova et al., 2017) (reverse mutations (Domchek, 2017) or gene fusion under the transcriptional control of heterologous promoter (Ter Brugge et al., 2016)) and suppression of NHEJ (caused by specific 53BP1 mutation in BRCA1 protein sequence) (Bouwman et al., 2010) (Hurley et al., 2019).

Downregulation of poly (ADP-ribose) glycohydrolase (PARG) protein levels is another hypothesized mechanism of PARPi resistance. Some studies suggest that depletion of PARG leads to PARPi resistance in BRCA2-deficient mouse mammary tumor models, which results in increased PAR levels even when PARP1 is largely or partially inhibited, thus counteracting PARP1 trapping and promoting PARPi resistance (Miwa et al., 1974) (Gogola et al., 2018). Moreover, miRNA expression patterns (especially miRNA-622 level) and drug efflux are also known to act as resistance mechanisms in PARPi therapy (Choi et al., 2016) (Rottenberg et al., 2008) (Vaidyanathan et al., 2016).

## Immune checkpoint inhibitors—In brief

The observed resistance toward PARPi triggered development of strategies combining PARPi with other therapies. The beforementioned mechanism of PARP trapping is known to sensitize cells to an alkylating agent temozolomide, while the catalytic inhibition enhances the effect of topoisomerase inhibitors (Murai et al., 2012) (Murai et al., 2014). The results of combining chemotherapy with PARPi are confounding, as the dose-limiting tissue toxicity is often reported. Also, due to the overlapping mechanisms of action, the mechanisms of resistance can also be shared and become a prominent limiting factor. This top of the iceberg of mechanistic complexity of action of PARPi and chemotherapy agents resulted in relatively slow development of combination therapies (Dréan et al., 2016) (Lu et al., 2018). Despite this, a recent meta-analysis shows a promising, yet cautious, view on this topic (Ren et al., 2021).

**FIGURE 3:**
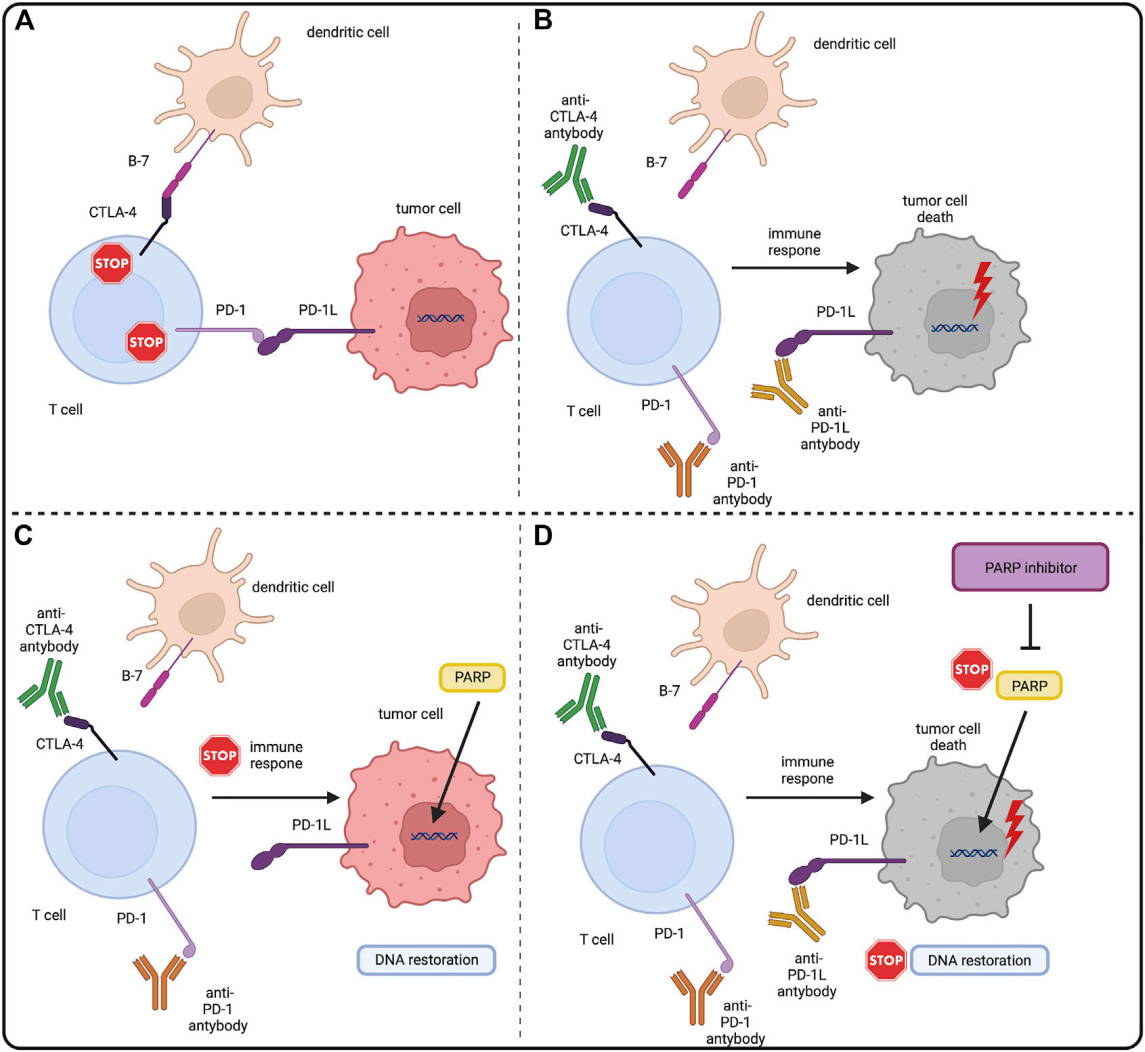
PARP inhibitors and immune checkpoint inhibitors–molecular rationale. **(A)** CTLA4 and PD1 are surface molecules present on T-cells. CTLA4 binds with the B7 molecule and PD1 binds with PD-L1 present on antigen presenting cells (APCs) and tumor cells. This downregulates the immunogenic response of both T-cells, allowing tumor cells to grow. **(B)** Immune checkpoint inhibitors (ICIs) by binding with CTLA4 and PD(L)1 block the downregulation of T-cells allowing them to eliminate tumor cells with the immune response. Also tumor cells genome instability makes them also more prone to the tumor mutation burden (TMB) and mutation overload leads to their death. **(C)** PARP role in DNA restoration allows tumor cells to repair DNA lesions and mitigate the TMB, eventually enabling them to evade the immune response. **(D)** PARP inhibitors by blocking the PARP DNA repair pathways in tumor cells stop the DNA restoration processes, exposing tumor cells to the immune response of T-cells under the treatment of ICIs. Created with BioRender.com.

An interesting direction of development of new combined therapies seems to be combination of PARPi with immunotherapies, one of the most promising being immune checkpoint inhibitors (ICIs). ICIs block surface proteins: T lymphocyte-associated antigen-4 (CTLA4) and programmed cell death receptor-1 (PD1) expressed by activated T-cells, and its ligand PD-L1 (Carlino et al., 2021). The mechanism of action of ICIs is based on enhancement of the immune response against cancer, namely the activation of T cells, which are stimulated by their surface receptors TCR and a costimulatory signal provided by CD28 (Lucas et al., 1995). PD-1 and CTLA4 are vital transmembrane receptor proteins engaged in the downregulation of T cells. PD1 binds to its PD-L1 ligand, which is present on tumor cells and antigen-presenting cells, causing a cascade of intracellular reactions leading to inactivation of the CD28 protein and inhibition of T cell activation (Walunas et al., 1996) (Brown et al., 2003) (Ishida et al., 2002) (Freeman et al., 2000). The anti-stimulatory effect of CTLA4 is slightly weaker. It is based on the competitive binding of CD28 ligands—B7-1 (CD80) and B7-2 (CD86) - located on antigen presenting cells (APC) with higher affinity (Peeraphatdit et al., 2020) (Peyraud and Italiano, 2020) (Wu et al., 2021) (Walunas et al., 1994) (Schweitzer and Sharpe, 1998). Malignant cells can create immunosuppressive tumor microenvironment (TME). TME arising relates to recruitment of regulatory T-cells (Treg) (Li et al., 2020). In TME PD1 and CTLA4 on T-cells and PD-L1 on cancer cells expression is upregulated. That prevents the effective anti-tumor immune response. Blocking these molecules by ICIs allows eliminating local suppression and inducing cancer-cell killing by CD8 positive T cells producing interferon gamma (IFN-γ) and tumor necrosis factor α (TNF-α) (Carlino et al., 2021) (Rameshbabu et al., 2021). The simplified rationale for combination of PARPi and ICIs was shown in Figure 3.

The application of ICIs into clinical cancer immunotherapy has a huge impact on patients' life and cancer response to applied therapy. Since 2011, many ICIs have been approved by FDA. Presently available ICIs are targeting CTLA4 (ipilimumab), PD1 (nivolumab, pembrolizumab, camipilimab) and PD-L1 (atezolizumab, avelumab, durvalumab). Their indications include melanoma (Carlino et al., 2021) (Larkin et al., 2015), renal cell carcinoma, non-small cell lung carcinoma (NSCLC), head and neck squamous cell carcinoma (HNSCC), metastatic urothelial carcinoma, gastric cancer, metastatic triple negative breast cancer, hepatocellular carcinoma (HCC), solid tumors, Merkel cell carcinoma, colorectal cancer, classical Hodgkin’s lymphoma. Many clinical trials are currently underway, so the list of approved drugs and their indications can be expected to expand (Twomey and Zhang, 2021) (Lee et al., 2022). (Supplementary Table S2)

Combining PARPi therapy with agents interacting with PD1/PD-L1 pathways was based on observations that DNA-damaging agents lead to the activation of interferon pathways due to DNA damage (Bakhoum et al., 2018). It has also been observed that the level of interferon expression has an impact on levels of PD-L1 (Garcia-Diaz et al., 2017) and that PARPi themselves cause upregulation of PD-L1 (Jiao et al., 2017). The combination of these three pieces of information suggests the potential of combining PARP inhibitors as a DNA-damaging agent and drugs interacting with PD1/PD-L1 pathways. Moreover, numerous mutations occurring in tumor cells, particularly the non-synonymous single nucleotide variants (nsSNVs), inevitably lead to the tumor mutation burden (TMB) and may lead to the increase of immunogenic peptides, which are known to be significantly correlated with the ICI response (Snyder et al., 2014) (Rizvi et al., 2015) (Hellmann et al., 2018). TMB is also thought to be correlated with the neoantigen load on the tumor cells, being an important predictive factor of the therapeutic response of ICIs (Schumacher and Schreiber, 2015) (Lee et al., 2018) (McGranahan et al., 2016). Highly mutated tumors often exhibit deficiencies in DDR pathways, which are also suggested to be closely related to the TMB (Mouw et al., 2017). Thus, a possible novel therapeutic approaches that combine ICIs with DDR blocking agents have been considered. Here we focus on the combination of ICIs and PARPi, the latter being one of the most effective agents in their field.

## Combination of PARPi and anti-PD1/PD-L1 ICIs—Clinical trials

Based on the promising results of various preclinical studies providing a rationale for combining PARPi with immunotherapy in cancer patients, (Jiao et al., 2017) (Wang et al., 2019b) (Ding et al., 2018) (Pantelidou et al., 2019) (Shen et al., 2019), several clinical trials have been conducted (Table 3). A significant group of the available clinical trials focused on non-small cell lung carcinoma (NSCLC). A study, evaluating dose and safety of veliparib combined with nivolumab and platinum doublet chemotherapy (pemetrexed and paclitaxel) focused on patients with metastatic and advanced NSCLC [NCT02944396]^1^. The overall response rate (ORR) reached 27% for cohort with pemetrexed and 17% for paclitaxel. This trial also confirmed the anticipated safety signals with no additional toxicity upon adding veliparib to these regimens (Clarke et al., 2021).

**TABLE 3:** List of selected PARP inhibitors and immune checkpoint inhibitors combination therapies. Full access to the research studies description is available on ClinicalTrials.gov.

PARP inhibitor	Immune checkpoint inhibitor	Tumor	Phase	Status	Trial ID (NCT number)
Olaparib	Tremelimumab	Platinum-sensitive advanced epithelial ovarian, fallopian tube, or primary peritoneal carcinoma	2	Active, not recruiting	NCT04034927
	Pembrolizumab	Recurrent or metastatic squamous cell carcinoma of head and neck	2	Recruiting	NCT04643379
		Advanced pancreatic cancer patients with germline BRCA1/2m	2	Recruiting	NCT04548752
		Recurrent or metastatic cervical cancer patients who had disease progression during or after platinum-based chemotherapy	2	Active, not recruiting	NCT04641728
		Advanced HER2-negative breast cancer with various DDR mutations	2	Not yet recruiting	NCT05033756
		Advanced, metastatic melanoma with the genetic HR-mutation	2	Recruiting	NCT04633902
		Advanced BRCAm or homology-directed repair-defect breast cancer	2	Recruiting	NCT03025035
		Metastatic pancreatic adenocarcinoma with high tumor mutation burden	2	Not yet recruiting	NCT05093231
		HR-mutated or HRD-positive advanced or metastatic solid tumors	2	Recruiting	NCT04123366
		Platinum-sensitive recurrent ovarian cancer	2	Recruiting	NCT05158062
		Recurrent/metastatic, platinum resistant nasopharyngeal cancer	2	Recruiting	NCT04825990
		Newly diagnosed treatment-naïve limited-stage small cell lung cancer	3	Recruiting	NCT04624204
		Locally advanced or metastatic gastric carcinoma	2	Recruiting	NCT04209686
		Metastatic triple-negative breast cancers	2	Recruiting	NCT04683679
		BRCA non-mutated patients with platinum-sensitive recurrent ovarian cancer	2	Enrolling by invitation	NCT04361370
		Locally advanced or metastatic cholangiocarcinoma	2	Recruiting	NCT04306367
		Advanced or recurrent cervical carcinoma after standard chemotherapy	2	Recruiting	NCT04483544
		Unresectable, locally advanced, stage III NSCLC	3	Recruiting	NCT04380636
		Untreated metastatic pancreatic ductal adenocarcinoma	2	Recruiting	NCT04753879
	Atezolizumab	Locally advanced unresectable and or metastatic HER-negative breast cancer	2	Active, not recruiting	NCT02849496
	Durvalumab	Prior to primary debulking surgery in high-grade epithelial ovarian cancer	2	Recruiting	NCT04644289
		Maintenance therapy in BRCAwt recurrent ovarian cancer	2	Recruiting	NCT04742075
		IDH-mutated solid tumors (glioma, cholangiocarcinoma, and solid tumors)	2	Recruiting	NCT03991832
		DDR-mutated castration sensitive biochemically recurrent non-metastatic prostate cancer	2	Active, not recruiting	NCT03810105
		Biochemically recurrent prostate cancer in men predicted to have a high neoantigen load	2	Recruiting	NCT04336943
		EGFR-mutated adenocarcinomas that transform to SCLC and other neuroendocrine tumors	2	Recruiting	NCT04538378
		Advanced epithelial ovarian cancer in relapse	2	Active, not recruiting	NCT04015739
		Newly diagnosed advanced or recurrent endometrial carcinoma	3	Recruiting	NCT04269200
		Locally advanced or metastatic ER positive HER2 negative breast cancer	2	Recruiting	NCT04053322
		Stage IV NSCLC	2	Active, not recruiting	NCT03775486
		Newly diagnosed advanced ovarian, fallopian tube or primary peritoneal carcinoma or carcinosarcoma	3	Recruiting	NCT03737643
		Recurrent, persistent or metastatic endometrial cancer	2	Recruiting	NCT03660826
		Renal cell cancer	2	Recruiting	NCT03741426
		Platinum-resistant recurrent epithelial ovarian cancer, primary peritoneal or fallopian cancer with prior bevacizumab treatment	2	Recruiting	NCT04739800
		Platinum-resistant recurrent ovarian cancer	2	Recruiting	NCT03699449
		NSCLC patients who progressed on an anti-PD1/PD-L1 containing therapy	2	Recruiting	NCT03334617
		Advanced soft tissue sarcoma	3	Recruiting	NCT03784014
		Breast cancer	2	Recruiting	NCT01042379
	Tremelimumab + durvalumab	HR-mutated advanced or metastatic solid tumors (breast, lung, head and neck, clear cell renal, endometrial, ovarian, urothelial and prostate cancer	2	Recruiting	NCT04169841
Niraparib	Cetrelimab	Aggressive variant prostate cancers	2	Recruiting	NCT04592237
	Dostralimab	Metastatic, PD-L1-negative or immunotherapy-refractory triple-negative breast cancer	2	Recruiting	NCT04837209
		Small cell lung cancer (SCLC) and other high-grade neuroendocrine carcinomas	2	Recruiting	NCT04701307
		Recurrent or metastatic head and nead squamous carcinoma	2	Recruiting	NCT04313504
		Recurrent or progressive cervix cancer	2	Recruiting	NCT04068753
		Relapsed epithelial ovarian cancer after treatment with PARPi	2	Not yet recruiting	NCT05126342
		Advanced NSCLC and/or malignant pleural mesothelioma, and positive for PD-L1 expression and germline or somatic mutations in the HR genes	2	Recruiting	NCT04940637
		HPV-negative squamous cell carcinoma of head and neck	2	Recruiting	NCT04681469
		Recurrent or primary advanced endometrial cancer	3	Active, not recruiting	NCT03981796
		Metastatic or recurrent endometrial or ovarian carcinosarcoma	2,3	Recruiting	NCT03651206
		Relapsed malignant mesothelioma	2	Recruiting	NCT03654833
		Ovarian cancer progressing post-PARPi	2	Not yet recruiting	NCT05065021
	Pembrolizumab	Advanced or metastatic NSCLC following completion of standard of care first-line platinum-based induction chemotherapy with pembrolizumab	3	Recruiting	NCT04475939
	Atezolizumab	Recurrent ovarian, tubal or peritoneal cancer	3	Active, not recruiting	NCT03598270
	Sintilimab	Rare tumors	2	Not yet recruiting	NCT04423185
Talazoparib	Nivolumab	BRCA- or BRCAness-mutated resectable or metastatic melanoma	2	Recruiting	NCT04187833
	Atezolizumab	Advanced cancer	2	Recruiting	NCT02693535
	Avelumab	Locally advanced or metastatic clear-cell renal cell carcinoma	2	Recruiting	NCT04068831
		Locally advanced/metastatic urothelial carcinoma	2	Recruiting	NCT04678362
		Recurrent or persistent endometrial cancer	2	Recruiting	NCT02912572
		Epithelial ovarian cancer	3	Recruiting	NCT05059522
Rucaparib	Nivolumab	Refractory leiomyosarcoma	2	Active, not recruiting	NCT04624178
		Advanced or metastatic cholangiocarcinoma	2	Recruiting	NCT03639935
		Relapsed ovarian, fallopian tube or peritoneal cancer	2	Recruiting	NCT02873962
		Platinum-sensitive SCLC	2	Recruiting	NCT03958045
	Atezolizumab	DDR-deficient or platinum sensitive solid tumors	2	Recruiting	NCT04276376

PD-L1 is known target for most of the ICIs in use as monotherapies, therefore the impact of PD-L1 expression differences between individuals on the trials involving ICIs and PARPi was probed in an interventional JASPER phase II study [NCT03308942]^2^ tested the combination of niraparib and pembrolizumab or dostarlimab on a group of chemotherapy-naïve patients with locally advanced or metastatic NSCLC with no prior PD-(L1) chemotherapy. The group was divided into two cohorts regarding the PD-L1 expression status of patients: PD-L1-rich (tumor proportion score–TPS ≥ 50%) and PD-L1-poor (TPS <50%). The ORR, duration of response (DOR), progression-free survival (PFS), and safety were assessed as endpoints. The study demonstrated that the combination of niraparib and pembrolizumab induces durable responses in patients with NSCLC, with larger effects in the PD-L1-rich cohort. Moreover, the combination showed no new safety signals (Ramalingam et al., 2022). As this combination was shown to be active and well tolerated, the ZEAL-1L phase III study [NCT04475939]^3^ was launched to compare the efficacy and safety of maintenance of niraparib + pembrolizumab versus pembrolizumab + placebo in patients with NSCLC (Ramalingam et al., 2021).

Nonetheless, the combination of another PARPi, olaparib, and durvalumab in a phase II study [NCT02484404]^4^ applied to patients with relapsed SCLC did not meet the present bar for efficiency. The tumor responses were predicted by the preexisting TILs level, which suggests an immune-mediated response as a predictive marker. Therefore, identification of patients with inflamed phenotype at the baseline may help to identify those most likely to respond to ICIs, although the predictive value of the preexisting CD8^+^ T-cell infiltrates must be confirmed in larger cohorts (Thomas et al., 2019).

Although in case of prostate cancer, ICIs have been shown to be ineffective as single agents, the results from a cohort analysis of the phase II CheckMate 9KD trial [NCT03338790]^5^ suggest that the treatment with nivolumab plus rucaparib may produce positive results in patients with HRD-positive chemotherapy naive metastatic castration-resistant prostate cancer (mCRPC). However, there was limited clinical activity of the combination therapy in patients with HRD-negative tumors. The confirmed ORR among the patients with HRD-positive tumors was 25% in comparison with 5.3% for the HRD-negative patients (Fizazi et al., 2022).

Moreover, the combination of olaparib (agent that demonstrated an improvement in median PFS in patients with prostate cancer) and durvalumab evaluated in the castration-resistant prostate cancer in a phase I/II MEDI4736 study [NCT02484404] showed eight out of 17 patients exhibited radiographic and/or PSA responses. The efficacy was noted particularly in men with DDR abnormalities (12-month PFS probability of DDR-deficient reached 83,3%, vs. 36,4% in DDR-proficient patients). Those with fewer peripheral myeloid-derived suppressor cells (pMDSC) were also more likely to respond. This suggests DDR deficiency and pMDSC level as predictive markers of the response (Karzai et al., 2018).

In order to identify biomarkers of a promising response to a combined PARPi and ICI, immunogenomic profiling and single-cell imaging have been performed on tumor samples subjected to such regimens. In a phase I/II trial [NCT02657889]^6^ of niraparib and pembrolizumab in ovarian cancer two determinants of response were identified: mutational signature 3 (correlating with the HR in DDR), and positive immune score as a function of interferon-primed exhausted CD8^+^ T cells in the tumor microenvironment. The interactions of exhausted CD8^+^ T-cells, PD-L1+ macrophages, and PD-L1 tumor cells with each other were noted in the single-cell spatial analysis, and PD-L1 tumor cells were shown to be the mechanistic response determinants, confirming the observations from previous studies (Färkkilä et al., 2020).

Patients with various solid tumors, including ovarian, breast and gastric cancer, were investigated in a phase I/II study MEDIOLA [NCT02734004]^7^ evaluating the effect of the combination of olaparib and durvalumab. In a germline *BRCA1/2*m metastatic breast cancer group, 80% of patients had disease control at 12 weeks and 50% at 28 weeks. Higher ORR and longer overall survival (OS) were observed in patients who had no prior line of chemotherapy in comparison to those with two prior lines (78% ORR and 21,3 months OS vs. 50% ORR and 16,9 months OS respectively). The investigated combination of agents exhibited promising activity and safety consistent with the profiles of individual agents (Domchek et al., 2020). In another cohort of this study enrolling patients with germline *BRCA1/2*m platinum-sensitive relapsed ovarian cancer, showed ORR of 63% and a 12-week DCR of 81%. The combination was well tolerated and the tumor responses in this initial analysis were higher in comparison with those reported for single-agent therapy with PARPi (Drew et al., 2018). However the results of another cohort of this study compassing patients with relapsed gastric cancer were less promising. The ORR was 10% and the disease control rate (DCR) at 26%. The combination was tolerable, with no unexpected adverse events. The durable responses after the combination of olaparib and durvalumab suggest synergistic treatment effect of the combination in some patients. Nonetheless, DCR value did not meet the target because of the high rate of early progressive diseases (PDs) occuring after the olaparib run in. Therefore, due to the initial treatment failures, an addition of new more effective therapies to the combination should be taken into account (Bang et al., 2019).

Besides this, several different phase I/II clinical trials were performed to verify the safety, toxicity and tolerability of the combination of PARPi and ICIs on patients with advanced solid tumors. In phase Ib IOLite study [NCT03307785]^8^ dostarlimab in combination with niraparib and niraparib + bevacizumab was shown to be well tolerated. Besides this, the study also evaluated combination of chemotherapy with PARPi and ICI, also with good tolerability. None of the combination agents used in this study altered the pharmacokinetics of dostarlimab nor niraparib. Moreover, no new safety signals were noted (Gabrail et al., 2019). In another phase I/II TOPACIO trial [NCT02657889]^9^ therapy combining niraparib and pembrolizumab in patients with ovarian and triple negative breast carcinoma showed general clinical improvement. However, the ovarian carcinoma cohort did not meet the primary endpoint with ORR of 18%, and the median duration of the response was not reached as well (Konstantinopoulos et al., 2019). The significantly higher response rates in patients with *BRCA1/2*m tumors was observed only in the breast cancer arm, with ORR of *BRCA1/2*m of 74% vs. wt*BRCA1/2* 11%. The PFS and DCR were 8.3 months and 80% for *BRCA*m, and 2.1 month with 33% for wt*BRCA1/2*, respectively (Vinayak et al., 2019). Finally, an ongoing JAVELIN PARP Mendley phase Ib/II study [NCT03330405]^10^ enrolled patients with advanced breast cancer cohorts treated with avelumab plus talazoparib. It showed preliminary antitumor activity and safety profile comparable to that of these agents used as monotherapies (Yap et al., 2020).

The clinical trials regarding combined therapies of PARPi and ICIs on patients with advanced solid tumors comprise also a novel agents. Pamiparib is an experimental selective PARP1/2i recently approved in China for the treatment of germline *BRCA1/2*m-associated recurrent advanced ovarian, fallopian tube or primary peritoneal cancer previously treated with two or more lines of chemotherapy [NCT03333915]^11^ (Markham, 2021). The safety of combination of pamiparib and tiselizumab was explored in a phase Ia/b trial [NCT02660034]^12^ on patients with advanced solid tumor. The combination treatment achieved an ORR of 20% and was well-tolerated, although the higher rate of immune-related hepatitis was noted in 8% of patients (Friedlander et al., 2019).

Even though the standard of care for muscle-invasive bladder cancer (MIBC) remains the relatively curative radical cystectomy, new non-surgery treatment approaches, including PARPi and ICIs, are being developed. The preliminary data from the phase II NEODURVARIB trial [NCT03534492]^13^ suggest that durvalumab in combination with olaparib administered prior to the surgery could be active and tolerated neoadjuvant treatment for MIBC, with the pathological complete response rate of 44,5% (Rodriguez-Moreno et al., 2020).

Numerous clinical trials are still ongoing in a vast range of cancers that will help to probe the features of the PARPi plus anti-PD-(L)1 combination therapy.

## Combination of PARPi and CTLA4 inhibitors—Clinical trials

In contrast to the anti-PD(-L)1 combination, which is investigated with much attention, the combination therapies of anti-CTLA4 and PARPi are largely unexplored. Despite the fact that previous studies demonstrated that tumors harboring BRCA1/2 dysfunction and treated with PARPi could increase tumor immunogenicity, therefore sensitizing tumor cells to anti-CTLA4 agents, the whole process remains understudied (Snyder et al., 2014) (Clarke et al., 2009) (McAlpine et al., 2012) (Wen and Leong, 2019) (Brown et al., 2016) (Higuchi et al., 2015).

Nonetheless, few important trials are currently conducted. The combination of olaparib and tremelimumab was verified in a phase I/II study [NCT02571725]^14^ involving women with BRCA1/2-deficient recurrent ovarian cancer. The preliminary results demonstrated a significant therapeutic effect along with acceptable tolerability (Adams et al., 2017).

This combination of olaparib plus tremelimumab is also under investigation in an ongoing phase II trial for patients with recurrent ovarian, fallopian tube or peritoneal cancer [NCT04034927]^15^.

In an ongoing phase II study [NCT04169841]^16^ the efficacy of olaparib combined with double immunotherapy of durvalumab and tremelimumab is being evaluated on patients with solid cancers, who were selected for this study based on their HR repair mutation profile and response after a previous olaparib treatment. This is the first clinical trial evaluating the combination of PARPi with ICI double therapy. Nonetheless, the combination of durvalumab plus tremelimumab itself has been studied before. To date, it has not shown significant advantages in comparison with the durvalumab monotherapy but displayed certain advantages over the traditional chemotherapies in some tumors, although more data of higher quality is required in this area (Arru et al., 2021).

Despite a relative shortage of published results regarding the combination of PARPi and anti-CTLA4 antibodies, the ongoing clinical trials may help to revive the promising antitumor activity of this approach.

## PARP inhibitors—Diagnostic tools

The successful introduction of PARPi into current oncology treatment methods causes an urgent need to develop modern techniques, which can help to predict tumor sensitivity to this type of therapy. A useful role in the selection process could be played by some specific biomarkers.

The most predictive marker at present are *BRCA1/2*m (Ganguly et al., 2016). Latest research studies demonstrated that a general testing of ovarian cancer patients for the presence of *BRCA1/2*m could be useful in planning anticancer therapy (Vos et al., 2020). Prediction of PARPi sensitivity is facilitated by several interesting approaches. One of them is BRCAnalysis CDx, which offers the analysis of germline*BRCA1/2*m–unfortunately, this method does not measure HR deficiency and might miss some of the patients who could have benefited from PARPi treatment (Gunderson and Moore, 2015). Another one is FoundationOne Liquid CDx, which offers tests of 324 genes including *BRCA1* and *BRCA2* (Woodhouse et al., 2020). Unfortunately, the status of *BRCA1/*does not always correlate with tumor sensitivity to PARPi therapy (Jonsson et al., 2019). Luckily, some other mutations have been described as potential biomarkers to predict therapeutic efficiency (Criscuolo et al., 2019). Also, a HR deficiency score was presented as a possible biomarker of chemotherapy efficacy in some tumors (Telli et al., 2016), and it could conceivably be extended to predicting the possible cancer response to PARPi therapy. Furthermore, specific methylation patterns could also be helpful in prediction of therapy effectiveness. Recent research studies presented hypermethylation of *RAD51* (Kondrashova et al., 2018) or hypermetylation of *HOXA9* (Montavon et al., 2012) as such promising markers.

Other potentially valuable approaches are focused on the assessment of the expression levels and modulation of various PARP1 regulators. One of these proteins is HPF1. Depletion of this protein makes PARPi more effective and induces tumor cell sensitivity to treatment with PARPi and other DNA-interacting drugs (Gibbs-Seymour et al., 2016). Another one is Y-box-binding protein (YB1), which inhibits PAR degradation by PARG (Alemasova et al., 2016). YB1 was presented as a protein playing a crucial role in tumor cell chemoresistance and a beneficial role of combining therapy with PARP inhibitors and DNA damaging agents was suggested as a potential answer to YB1 activity (Alemasova et al., 2018). The assessment of the YB1 level in patients with tumors could also be used as a promising biomarker in PARPi treatment. Moreover, depletion of PARP1-interacting Src-associated substrate during mitosis 68 kDA (Sam68), leads to impaired PARP1 activation, establishing Sam68 as a potential PARP1 activator (Fu et al., 2016). Using Sam68 as a biomarker could boost potential cancer therapy (Zhang et al., 2015) (Zhang et al., 2009). The other tested proteins were Barrier to Autointegration Factor 1 (Banf1)—a negative regulator of PARP1 activity (Bolderson et al., 2019) and TRIP12—ubiquitin E3 ligase, which regulates PARP1 stability (Gatti et al., 2020). These two proteins could also play an important role in the prediction of success in PARPi therapy and need to be studied further.

## Future perspectives and limitations

Recent years have brought plethora of studies exploring the combination of PARPi with other agents. The efficacy of the combination therapy of PARPi and ICIs, which we discussed in this review, is being tested in numerous clinical trials.

Nonetheless clinical trials themselves are not fully conclusive. Most of them rely on ORR or DCR, the early endpoints, which provide information only about the initial phase of therapy, while the prolonged monitoring data are still mostly undetermined. Moreover, all current clinical trials are non-randomized, allowing only cross-trial comparison. Thus, PARPi and ICI treatment strategies should be optimized by recruiting randomized controlled multi arms phase III trials, which are designed to enable the interpretation of the effect of each drug alone or in combination.

Another blind spot of clinical studies involving PARPi and ICIs is that most of the tumor types where the combination strategy was tested already had demonstrated significant improvement from PARPi monotherapy and limited benefit from ICI addition. Therefore, it would be reasonable to investigate the new combination of PARPi and ICIs in a group of patients that have an unmet anticancer clinical need, rather than in those with well-established therapies. Thus, studies involving patients who do not respond well to PARPi or ICI monotherapies should be taken into account. Finally, the schedule and timing of these studies should be optimized to preserve tolerability and the associated impact on health care costs should also be considered in terms of the therapy duration.

Although the mechanism of the combinatorial effect of PARPi and ICIs is now intensively investigated and several mechanisms have been proposed to be involved in this process in HR-deficient patients, like those with BRCA1/2 dysfunction, the rationale for using this therapy on patients with functional HR mechanisms is yet to be determined. Uncovering the rationale of this combining therapy in non-HRD patients and more preclinical *in vivo* trials will be crucial to pick up the target group of patients that would benefit most of the therapy.

The crucial factor in identifying the optimal target population of patients are biomarkers. Although in tumors with *BRCA1/2*m or HRD the effect is beneficial, HR-proficient patient markers are yet to be determined. Elucidating the molecular profile of this population will allow answering the question whether the tumors could be sensitized to ICIs by PARPi. The deeper understanding of molecular mechanisms of PARPi and ICI pathways should result in a collection of biomarkers that could help to understand the potential sensitivity and resistance.

## Conclusion

PARPi monotherapy has proved to be a milestone in the treatment of many *BRCA1/2*m cancers, bringing patients hope of an effective therapy. Throughout the years many therapies combining PARPi with different agents were proposed, ICIs being the promising among them, Currently, numerous clinical trials are ongoing, validating the combined therapies with PARPi and ICIs. Although the studies do not encompass the whole complexity of these agents, the available data is promising. The key to comprehend the true power of PARPi- ICI combination is an in-depth understanding of their molecular mechanism. Identifying such biomarkers might also facilitate the search for adequate biomarkers that could help to guide the doctor’s hand to treat the patients in the most effective way and find the most suitable place for the combination of PARPi and ICIs in the clinic.

## Footnotes

1 Phase 2, Multi-Arm Study of Niraparib Administered Alone and in Combination With a PD-1 Inhibitor in Patients With Non-Small Cell Lung Cancer. Available online at: https://clinicaltrials.gov/ct2/show/NCT03308942 (accessed 17 October 2022).

2 Phase 2, Multi-Arm Study of Niraparib Administered Alone and in Combination With a PD-1 Inhibitor in Patients With Non-Small Cell Lung Cancer, Available online at: https://clinicaltrials.gov/ct2/show/NCT03308942 (accessed 17 October 2022).

3 Phase 3, Randomized, Double-Blind, Placebo-Controlled, Multicenter Study Comparing Niraparib Plus Pembrolizumab Versus Placebo Plus Pembrolizumab as Maintenance Therapy in Participants Whose Disease Has Remained Stable or Responded to First-Line Platinum Based Chemotherapy With Pembrolizumab for Stage IIIB/IIIC or IV Non-Small Cell Lung Cancer (ZEAL-1L). Available online at: https://clinicaltrials.gov/ct2/show/NCT04475939 (accessed 17 October 2022).

4 Phase I/II Study of the Anti-Programmed Death Ligand-1 Antibody Durvalumab (MEDI4736) in Combination With Olaparib and/or Cediranib for Advanced Solid Tumors and Advanced or Recurrent Ovarian, Triple Negative Breast, Lung, Prostate and Colorectal Cancers, Available online at: https://www.clinicaltrials.gov/ct2/show/NCT02484404 (accessed 17 October 2022).

5 A Phase 2 Study of Nivolumab in Combination With Either Rucaparib, Docetaxel, or Enzalutamide in Men With Castration-resistant Metastatic Prostate Cancer. Available online at: https://clinicaltrials.gov/ct2/show/NCT03338790 (accessed 17 October 2022).

6 Phase 1/2 Clinical Study of Niraparib in Combination With Pembrolizumab (MK-3475) in Patients With Advanced or Metastatic Triple-Negative Breast Cancer and in Patients With Recurrent Ovarian Cancer. Available online at: https://clinicaltrials.gov/ct2/show/NCT02657889 (accessed 17 October 2022).

7 A Phase I/II Study of MEDI4736 (Anti-PD-L1 Antibody) in Combination With Olaparib (PARP Inhibitor) in Patients With Advanced Solid Tumors. Available online at: https://clinicaltrials.gov/ct2/show/NCT02734004 (accessed 17 October 2022).

8 Phase 1b Dose-Finding Study of Niraparib, TSR-022, Bevacizumab, and Platinum-Based Doublet Chemotherapy in Combination With TSR-042 in Patients With Advanced or Metastatic Cancer. Available online at: https://www.clinicaltrials.gov/ct2/show/NCT03307785 (accessed 17 October 2022).

9 Phase 1/2 Clinical Study of Niraparib in Combination With Pembrolizumab (MK-3475) in Patients With Advanced or Metastatic Triple-Negative Breast Cancer and in Patients With Recurrent Ovarian Cancer. Available online at: https://clinicaltrials.gov/ct2/show/NCT02657889 (accessed 17 October 2022).

10 Phase 1/2 Clinical Study of Niraparib in Combination With Pembrolizumab (MK-3475) in Patients With Advanced or Metastatic Triple-Negative Breast Cancer and in Patients With Recurrent Ovarian Cancer. Available online at: https://clinicaltrials.gov/ct2/show/NCT02657889 (accessed 17 October 2022).

11 An Open Label, Multi-Center Phase I/II Study to Evaluate Efficacy and Safety of BGB-290 in Chinese Subjects With Advanced Ovarian Cancer, Fallopian Cancer, and Primary Peritoneal Cancer or Advanced Triple Negative Breast Cancer. Available online at: https://clinicaltrials.gov/ct2/show/NCT03333915 (accessed 17 October 2022).

12 A Phase 1/1b, Open Label, Multiple Dose, Dose Escalation and Expansion Study to Investigate the Safety, Pharmacokinetics and Antitumor Activity of the Anti-PD-1 Monoclonal Antibody BGB-A317 in Combination With the PARP Inhibitor BGB-290 in Subjects With Advanced Solid Tumors. Available online at: https://clinicaltrials.gov/ct2/show/NCT02660034 (accessed 17 October 2022).

13 Impact of the Combination of Durvalumab (MEDI4736) Plus Olaparib (AZD2281) Administered Prior to Surgery in the Molecular Profile of Resectable Urothelial Bladder Cancer. Available online at: https://clinicaltrials.gov/ct2/show/NCT03534492 (accessed 17 October 2022).

14 Phase 1-2 Study of the Combination of Olaparib and Tremelimumab, in BRCA1 and BRCA2 Mutation Carriers With Recurrent Ovarian Cancer. Available online at: https://clinicaltrials.gov/ct2/show/NCT02571725 (accessed 17 October 2022).

15 A Phase II Randomized Trial of Olaparib Versus Olaparib Plus Tremelimumab in Platinum-Sensitive Recurrent Ovarian Cancer. Available online at: https://clinicaltrials.gov/ct2/show/NCT04034927 (accessed 17 October 2022).

16 Precision Medicine Phase II Study Evaluating the Efficacy of a Double Immunotherapy by Durvalumab and Tremelimumab Combined With Olaparib in Patients With Solid Cancers and Carriers of Homologous Recombination Repair Genes Mutation in Response or Stable After Olaparib Treatment. Available online at: https://clinicaltrials.gov/ct2/show/NCT04169841 (accessed 17 October 2022).
