# Supplementary material for: The potential of PARP inhibitors in targeted cancer therapy and immunotherapy
Source: Front Mol Biosci. 2022 Dec 1;9:1073797. doi: 10.3389/fmolb.2022.1073797 (PMC9751342; doi:10.3389/fmolb.2022.1073797)
Supplement: Supplementary file 1 [file Table1.DOCX]

Supplementary Table 1. A list of selected PARP inhibitors adverse effects. Grade refers to the severity of an adverse event. Grade 1 - mild, grade 2 - moderate, grade 3 - severe, grade 4 -life threatening or disabling, grade 5 - death-related adverse event.

^[[1]](#footnote-1)^

|  | **Niraparib^[[2]](#footnote-2)^** | | **Olaparib^[[3]](#footnote-3)^** | | **Rucaparib^[[4]](#footnote-4)^** | | **Talazoparib^[[5]](#footnote-5)^** | |
| --- | --- | --- | --- | --- | --- | --- | --- | --- |
| **System organ class** | **All grades** | **Grade 3 or 4** | **All grades** | **Grade 3 or 4** | **All grades** | **Grade 3 or 4** | **All grades** | **Grade 3 or 4** |
| **Infections and infestation** | Very common: urinary tract infection | Uncommon: urinary tract infection, bronchitis |  |  |  |  |  |  |
|  | Common: bronchitis, conjuctivitis |  |  |  |  |  |  |  |
| **Blood and lymphatic system disorders** | Very common: thrombocytopenia, anaemia, neutropenia, leukopenia | Very common: thrombocytopenia, anaemia, neutropenia | Very common: anaemia, neutropenia, thrombocytopenia, leukopenia | Very common: anaemia | Very common: anaemia, thrombocytopenia, neutropenia | Very common: anaemia | Very common: thrombocytopenia, anaemia, neutropenia, leucopenia | Very common: thrombocytopenia, anaemia, neutropenia |
|  | Uncommon: pancytopenia, febrile neutropenia | Common: leukopenia | Common: lymphopenia | Common: neutropenia, thrombocytopenia, leukopenia | Common: leukopenoia, lymphopenia, febrile neutropenia | Common: thrombocytopenia, neutropenia, febrile neutropenia, leukopenia | Common: lymphopenia | Common: leucopenia, lymphopenia |
|  |  | Uncommon: pancytopenia, febrile neutropenia |  | Uncommon: lymphopenia |  | Uncommon: lymphopenia |  |  |
| **Immune system disorders** | Common: hypersensitivity | Cncommon: hypersensitivity | Uncommon: hypersensitivity, angioedema | Rare: hypersensitivity | Common: hypersensitivity | Uncommon: hypersensitivity |  |  |
| **Metabolism and nutrition disorders** | Very common: decreased appetite | Common: hypokalemia | Very common: decreased appetite | Uncommon: decreased appetite | Very common:decreased appetite, increased blood creatinine | Common: decreased appetite, dehydration | Very common: decreased appetite | Uncommon: decreased appetite |
|  | Common: hypokalemia | Uncommon: decreased appetite |  |  | Common: hypercholesterolaemia, dehydration | Uncommon: increased blood creatinine, hypercholesterolaemia, |  |  |
| **Psychiatric disorders** | Very common: insomnia | Uncommon: insomnia, anxiety, depression, confusional state |  |  |  |  |  |  |
|  | common: anxiety, depression, cognitive impairment |  |  |  |  |  |  |  |
|  | Uncommon: confusional state |  |  |  |  |  |  |  |
| **Nervous system disorders** | Very common: headache, dizziness | Uncommon: headache | Very common: dizziness, headache, dysgeusia | Uncommon: dizziness, headache | Very common: dysgeusia, dizziness | Uncommon: dysgeusia, dizziness | Very common: dizziness, headache | Uncommon: dizziness, headache |
|  | Common: dysgeusia |  |  |  |  |  | Common: dysgeusia |  |
|  | Rare: Posterior Reversible Encephalopathy Syndrome (PRES) |  |  |  |  |  |  |  |
| **Cardiac disorders** | Very common: palpitations |  |  |  |  |  |  |  |
|  | Common: tachycardia |  |  |  |  |  |  |  |
| **Vascular disorders** | Very common: hypertension | Common: hypertension |  |  |  |  |  |  |
|  | Rare: hypertensive crisis |  |  |  |  |  |  |  |
| **Respirator, thoracic and mediastinal disorders** | Very common: dyspnoea, cough, nasopharyngitis | Uncommon: dyspnoea, epistaxis, pneumonitis | Very common: cough, dyspnoea | Common: dyspnoea | Common: dyspnoea | Uncommon: dyspnoea | Very common: dyspnoea, cough, nasopharyngitis |  |
|  | Common: epistaxis |  |  |  | Uncommon: cough |  |  |  |
|  | Uncommon: pneumonitis |  |  |  |  |  |  |  |
| **Gastrointestinal disorders** | Very common: nausea, constipation, vomiting, abdominal pain, diarrhoea, dyspepsia | Common: nausea, vomiting, abdominal pain | Very common: vomiting, diarrhoea, nausea, dyspepsia | Common: vomiting, diarrhoea, nausea | Very common: nausea, vomiting, diarrhoea, dyspepsia, abdominal pain | Common: nausea, vomiting, upper abdominal pain | Very common: vomiting, diarrhoea, nausea, abdominal pain | Common: vomiting, abdominal pain |
|  | Common: dry mouth, abdominal distension, mucosal inflammation, stomatitis | Uncommon: diarrhoea, constipation, mucosal inflammation, stomatitis, dry mouth | Common: stomatitis, upper abdominal pain | Uncommon: stomatitis, upper abdominal pain |  | Uncommon: dyspepsia | Common: stomatitis, dyspepsia | Uncommon: diarrhoea, nausea |
|  |  |  |  | Rare: dyspepsia |  |  |  |  |
| **Skin and subcutaneous tissue disorders** | Common: photosensitivity, rash | Uncommon: photosensitivity, rash | Common: rash | Uncommon: rash | Very common: photosensitivity reaction, rash | Uncommon: photosensitivity reaction, rash, rash maculo-papular, palmar- plantar erythrodysaesthesia syndrome | Very common: alopecia |  |
|  |  |  | Uncommon: dermatitis |  | Common: rash maculo-papular, palmar- plantar erythrodysaesthesia syndrome, erythema |  |  |  |
|  |  |  | Rare: erythema nodosum |  |  |  |  |  |
| **Musculoskeletal and connective tissue disorders** | Very common: back pain, arthralgia | Uncommon: back pain, arthralgia, myalgia |  |  |  |  |  |  |
|  | Common: myalgia |  |  |  |  |  |  |  |
| **General disorders and administration site conditions** | Very common: fatigue, asthenia | Common: fatigue, asthenia | Very common: fatigue | Common: fatigue | Very common: fatigue, pyrexia | Common: fatigue | Very common: fatigue | Common: fatigue |
|  | Common: oedema peripheral |  |  |  |  | Uncommon: pyrexia |  |  |
| **Investigations** | Common: gamma-glutamyl transferase increased, AST increased, blood creatinine increased, ALT increased, blood alkaline phosphatase increased, weight decreased | Common: gamma-glutamyl transferase increased, ALT increased | Common: blood creatinine increased | Rare: blood creatinine increased |  |  |  |  |
|  |  | Uncommon: AST increased, blood alkaline phosphatase increased | Uncommon: mean cell volume increased |  |  |  |  |  |
| **Neoplasms benign, malignant and unspecified (including cysts and polyps)** |  |  | Uncommon: myelodysplastic syndrome/ acute myeloid leukaemia | Uncommon: myelodysplastic syndrome/ acute myeloid leukaemia | Common: myelodysplastic syndrome/ acute myeloid leukaemia | Common: myelodysplastic syndrome/ acute myeloid leukaemia |  |  |
| **Hepatobiliary disorders** |  |  |  |  | Very common: increased alanine aminotransferase, increased aspartate aminotransferase | Common: increased alanine aminotransferase, increased aspartate aminotransferase |  |  |
|  |  |  |  |  | Common: increased transaminase | Uncommon: increased transaminases |  |  |

1. Cancer Therapy Evaluation Program, Common Terminology Criteria for Adverse Events, Version 3.0, DCTD, NCI, NIH, DHHS March 31, 2003 (http://ctep.cancer.gov), Publish Date: August 9, 2006 (accessed Sept 10, 2022) [↑](#footnote-ref-1)
2. European Medicines Agency Web site https://www.ema.europa.eu/en/documents/product-information/zejula-epar-product-information_en.pdf. Accessed Sept 10, 2022 [↑](#footnote-ref-2)
3. European Medicines Agency Web site https://www.ema.europa.eu/en/documents/product-information/lynparza-epar-product-information_en.pdf. Accessed Sept 10, 2022 [↑](#footnote-ref-3)
4. European Medicines Agency Web site https://www.ema.europa.eu/en/documents/product-information/rubraca-epar-product-information_en.pdf. Accessed Sept 10, 2022 [↑](#footnote-ref-4)
5. European Medicines Agency Web site https://www.ema.europa.eu/en/documents/product-information/talzenna-epar-product-information_en.pdf. Accessed Sept 10, 2022 [↑](#footnote-ref-5)
