# Supplementary material for: The potential of PARP inhibitors in targeted cancer therapy and immunotherapy
Source: Front Mol Biosci. 2022 Dec 1;9:1073797. doi: 10.3389/fmolb.2022.1073797 (PMC9751342; doi:10.3389/fmolb.2022.1073797)
Supplement: Supplementary file 2 [file Table2.DOCX]

Table 3. A list of selected immune checkpoint inhibitor monotherapy trials. Full access to the research studies description is available on ClinicalTrials.gov ^[[1]](#footnote-1)2^

| **Immune checkpoint inhibitor** | **Tumor** | **Phase** | **Status** | **Trial ID (NCT number)** |
| --- | --- | --- | --- | --- |
| Atezolizumab | Urinary tract squamous cell carcinoma | 2 | Recruiting | NCT05038657 |
|  | Squamous cell carcinoma of head and neck | 2 | Recruiting | NCT04939480 |
|  | Cutaneous melanoma | 1 | Recruiting | NCT04020809 |
| Avelumab | Hodgkin lymphoma | 2 | Active, not recruiting | NCT03617666 |
| Cemiplimab | Secondary angiosarcoma | 2 | Recruiting | NCT04873375 |
|  | Stage II-IV cutaneous squamous cell carcinoma of head and neck | 2 | Recruiting | NCT03565783 |
|  | Advanced cutaneous squamous cell carcinoma | 2 | Active, not recruiting | NCT02760498 |
| Durvalumab | advanced hepatocellular carcinoma | 2 | Recruiting | NCT04294498 |
|  | Stage IIIB-IV NSCLC | 2 | Active, not recruiting | NCT03620669 |
|  | NSCLC | 2 | Not yet recruiting | NCT05206812 |
| Ipilimumab | Urothelial carcinoma | 1 | Completed | NCT00362713 |
|  | Melanoma | Early 1 | Completed | NCT00972933 |
|  | Advanced malignant melanoma | 4 | Active, not recruiting | NCT02068196 |
|  | Prostate cancer | Early 1 | Completed | NCT02113657 |
| Nivolumab | Recurrent/metastatic carcinosarcoma | 2 | Recruiting | NCT05224999 |
|  | Renal cell cancer and NSCLC with pleural effusion | 2 | Recruiting | NCT04749602 |
|  | Cancers with MMR deficiency | 2 | Completed | NCT04439214 |
|  | Primary central nervous system lymphoma | 2 | Recruiting | NCT04401774 |
|  | Recurrent prostate cancer | 2 | Recruiting | NCT04019964 |
|  | Hodgkin lymphoma | 2 | Recruiting | NCT03337919 |
| Pembrolizumab | Colorectal cancer | 2 | Not yet recruiting | NCT05131919 |
|  | Cutaneous squamous cell carcinoma of the head and neck | 2 | Recruiting | NCT05025813 |
|  | Prostate cancer | 2 | Recruiting | NCT04009967 |
|  | Diffuse large B cell lymphoma | 2 | Recruiting | NCT03990961 |
|  | HER2-negative breast cancer | 2 | Recruiting | NCT03989089 |
|  | Metastatic or recurrent squamous cell carcinoma of head and neck | 2 | Recruiting | NCT03813836 |
|  | NSCLC | 2 | Recruiting | NCT03526887 |

1. <https://clinicaltrials.gov/> (accessed Sept 10, 2022) [↑](#footnote-ref-1)
